# Paralysis

**Published:** 1856-05

**Authors:** 


*Paralysis.*

We copy the following account of a case of this interesting and formidable disease, from a letter, recently written to us by A. W. Dewey, M.D., of Strawtown, Ind. It will be read with interest and profit.

We have recently treated several cases of paralysis, arising from different causes, or rather dependent on different pathological conditions, and will furnish the readers of the *Journal* with an account of them as soon as time will permit.

"During last summer, I had partly prepared a communication, describing a case of paraplegia, which was at that time on hand, and privately soliciting your advice thereon. It was one for which I felt much solicitude, the patient being the wife of an esteemed friend—a very amiable young lady of 24 years, the mother of one child, five months old at the period of her attack, July 2d, 1855. On the morning referred to, when attempting to rise, she experienced, as she expressed it, a 'sensation of numbness in her limbs,' (inf. extremities only.) Her husband having gone to the store, some twenty rods off, she managed to get her feet off the bed and to dress herself. Supposing that a little exercise would rid her of the numbness, she succeeded, by the aid of her hands, in getting from the bed, when, to her dismay, she found her limbs of no more use to support her, and capable of but little more feeling than the skirts of her garments—in short, she found herself upon the floor, and obliged to remain there till her husband's return. I was then called in—found her resting as comfortable as if in the best of health; no irregularity of functions manifest, and all the secretions apparently normal. The lower extremities, however, were in a state of almost complete paralysis, and as clearly beyond the influence of the will as if severed from the body. Slight tingling or pricking sensations were felt on pressure. The temperature was natural as the body. No premonition had been noticed, except a feeling of weakness in the back the day previously.

"After trying the remedies usually resorted to in cases of this kind, *i. e.*, irritation along the spinal column, cold bathing, blistering, and cupping, and finding the conditions the same, I explored the contents of my journals and standard works (so called) within my reach, without discovering anything that promised much encouragement—unless it was to come from the unexplored and illimitable estuaries of electrical power. Thither I directed my inquiries: a galvanometer was procured, and the current applied and caused to pass in all possible directions, but chiefly along the spine, from the neck to the sacrum, and down the extremities, graduating the power to the full extent, borne without severe suffering. The *cause*, I believed to lie entirely within the spinal canal; but, of its precise nature, I could only guess—whether mechanical pressure, by collected fluid, or œdema of the membranes, or osseous enlargements, or absolute innervation by the loss of dynamic power, I had no means of certainly telling. The galvanism was applied twice daily, for a few days, and then but once. In conjunction, I gave the following: Strychnine, 1-16 gr.; sulph., 3 grs.; camph., 1 gr.; every four hours—and applied the cups once every other day. The strychnine was gradually increased, until $\frac{1}{8}$ gr. was given, when its effects began to be manifest. It was then discontinued a few days, and again used. By the third day of this course, there was promise of improvement—a partial return of sensibility, and power of slightly flexing the right foot.

The course was closely adhered to, and the improvement was gradually extended to a favorable conclusion. The right extremity was first to show signs of returning vitality. By the end of the third week, she could stand by the aid of a staff or chair; by the end of the fifth, could stand alone; and by the seventh, able to resume in part the duties of the household. As the case began to improve, and other duties (by the increase of the business at that season) claimed my time and attention, I deferred finishing the original letter, and finally abandoned the intention of forwarding it. A review of my diary has again brought the subject to mind, and elicited this report, which I hope may be the cause of eliciting some remarks from your clearly practical pen. Heretofore, in such cases, recovery has been regarded as doubtful, and their management difficult. Nothing is laid down, that I have yet seen, sufficiently clear in diagnosis or definite in treatment, as the basis of a practice with even a probable certainty of success. Can you not give us something upon the subject, reliable and philosophical, as much so as your pathology of fever, when the duties of your station permit?"

